# Familiarity effects in EEG-based emotion recognition

**DOI:** 10.1007/s40708-016-0051-5

**Published:** 2016-04-29

**Authors:** Nattapong Thammasan, Koichi Moriyama, Ken-ichi Fukui, Masayuki Numao

**Affiliations:** 1 Institute of Scientific and Industrial Research (ISIR), Osaka University, Ibaraki-shi, Osaka 567-0047 Japan; 2 Department of Computer Science and Engineering, Nagoya Institute of Technology, Showa-ku, Nagoya, 466-8555 Japan

**Keywords:** Electroencephalogram, Music-emotion, Classification, Familiarity

## Abstract

Although emotion detection using electroencephalogram (EEG) data has become a highly active area of research over the last decades, little attention has been paid to stimulus familiarity, a crucial subjectivity issue. Using both our experimental data and a sophisticated database (DEAP dataset), we investigated the effects of familiarity on brain activity based on EEG signals. Focusing on familiarity studies, we allowed subjects to select the same number of familiar and unfamiliar songs; both resulting datasets demonstrated the importance of reporting self-emotion based on the assumption that the emotional state when experiencing music is subjective. We found evidence that music familiarity influences both the power spectra of brainwaves and the brain functional connectivity to a certain level. We conducted an additional experiment using music familiarity in an attempt to recognize emotional states; our empirical results suggested that the use of only songs with low familiarity levels can enhance the performance of EEG-based emotion classification systems that adopt fractal dimension or power spectral density features and support vector machine, multilayer perceptron or C4.5 classifier. This suggests that unfamiliar songs are most appropriate for the construction of an emotion recognition system.

## Introduction

Owing to the high temporal resolution and low cost of electroencephalography (EEG), it has been extensively used in recent attempts to detect emotional states due to its prominence in high temporal resolution but low cost. EEG and emotion correlation reported in numerous studies [1, 2] combined with computational modeling [3] enables possibility of automatically estimating emotional states. The use of musical excerpts as stimuli is considered to be a promising approach because music is understood to be capable of strongly eliciting various emotions [4]. However, very little is currently known about the subjective characteristics of human music perception.

Music experience can be influenced by cultural background, age, gender, training, and familiarity with the music [5]. Specifically, as listening to familiar music involves expectation and prediction based on prior knowledge to musical excerpts, a listener’s memory might play a crucial role in musical perception and can affect the emotional reaction. Recent studies have used various measuring tools to determine the relationship between music familiarity and physiological signals. An fMRI study revealed that a feeling of familiarity with music or odors induced activation in the deep left hemisphere, while a feeling of unfamiliarity induced activation in the right hemisphere [6]. Researchers concluded that it is possible to trigger neural processes specific to the feeling of familiarity regardless of the type of triggering stimuli via processes that are likely related to the semantic memory system. Another fMRI study reported the role of familiarity in the brain’s correlation of music appreciation and suggested that music familiarity is related to limbic, paralimbic, and reward circuitries [7]. Evidence from electrodermal activity studies demonstrates that certain levels of expectation and predictability caused by familiarity play an important role in the experience of emotional arousal in response to music [8]. In another study, musical melody familiarity was seen to be correlated with event-related potentials observed along the frontocentral scalp with melodies with a higher degree of familiarity producing more negative potentials [9]. The researchers suggested that the feeling of familiarity could be involved in the processing mechanism at the conceptual level. To the best of our knowledge, however, the effect of music familiarity on brainwave patterns has not yet been fully explored. Even though the past decade has seen a growing interest in the automatic detection of emotion using EEG, such studies have overlooked music familiarity effects; however, if music familiarity actually has an effect on brain signals, ignoring familiarity would degrade EEG-based emotion recognition.

In this study, we present the first attempt to investigate the neural correlates of music familiarity by focusing on the differences among brain responses engendered by music samples of varying levels of familiarity. We constructed a model to classify emotional response to musical material in a manner similar to conventional approaches with taking familiarity into account. In this study, we used two different datasets; one constructed from our experimental work, and one extracted from the database for emotion analysis using physiological signals (DEAP) [10], an existing affective EEG database that has been extensively used in recent years in affective computing research. The experiments that produced both datasets focused on self-emotion annotation approaches based on the assumption that the emotions incurred when experiencing music are subjective.

Importantly, the emotion produced when experiencing musical stimuli can change over time, especially when listening to long-duration music. Cortical activity alternation over time during long music exposure was found in a previous EEG study [2]. Consequently, recent research has emphasized the importance of taking into account the time-varying characteristics of emotion [11] and performing emotion recognition in a continuous paradigm [12]. In this study, we took the continuous emotion recognition into account by applying the technique of temporal segmentation to both datasets and employing temporal continuous emotion annotation in our experiment.

Human emotion can be systematically described through mapping into a corresponding two-dimensional arousal-valence emotion space in which valence is represented as a horizontal axis indicating positivity of emotion and arousal is represented as a vertical axis indicating activation level of emotions. This emotion model was originally proposed by Russell [13] and is still frequently used in affective computing research, as it has been found to be a simple but highly effective model [3, 5].

## Experimental data

### Our dataset

#### Experimental protocol

We recruited a homogeneous population of 15 healthy subjects between 22 and 30 years of age (mean = 25.52, SD = 2.14). All subjects were students of Osaka University and had a minimal formal musical education; informed consent was obtained from all individual subjects included in the experiment. Each subject was requested to select 16 musical excerpts from a 40-song MIDI library and to indicate their familiarity with each selected song on a scale ranging from 1 to 6, corresponding to lowest and highest familiarity, respectively. The subjects were instructed to select eight songs with which they felt familiar (i.e., having familiarity ranking of 4–6) and eight unfamiliar songs (familiarity ranking 1–3). To facilitate familiarity judging, our data collection software provided a function to play short (<10s) samples of songs to the subjects.

To reduce cognitive load due to emotion reporting, separate annotation sessions were conducted following music listening/EEG recording sessions. In the first listening phase, the selected songs were presented as synthesized sounds using the Java Sound API’s MIDI package^1^, with four of the selected familiar songs played first, followed by four of the unfamiliar songs, then the other four familiar songs, and finally the remaining unfamiliar songs. Each song was played for approximately 2 min and a 16 s silent rest was inserted between each musical excerpt to reduce any influence of the previous song.

After listening to the 16 songs and taking a short rest, each subject proceeded to the second phase, an emotion annotation session without EEG recording. Using the assumption that emotional response can change over the course of time during a music listening session, each subject was instructed to describe his/her emotional reactions to selected songs presented in the same order as in the previous phase using our developed software. Each subject described his/her changing emotions by continuously clicking on the corresponding point in an arousal-valence emotion space shown on a monitor screen. To facilitate reporting, a brief guideline to the emotion space was also provided throughout annotation session. Arousal and valence were recorded independently as numerical values ranging from –1 to 1. After providing an emotion annotation for each song, each subject was asked to confirm or change his/her familiarity with the song and indicate how confident, on a discrete scale ranging from 1 to 3, he/she was of the correspondence between the annotated emotions and the emotions perceived during the first listening phase.

#### EEG recording and preprocessing

In this experiment, a Waveguard EEG cap^2^, placed in accordance with the 10–20 international system and referenced to the Cz electrode, was used to record EEG signals at a sampling frequency of 250 Hz. Twelve electrodes (Fp1, Fp2, F3, F4, F7, F8, Fz, C3, C4, T3, T4, and Pz) located near the frontal lobe which is believed to play a crucial role in emotion regulation [14] were selected for analysis. The impedance of each electrode was kept below 20 k$\Omega$ throughout the experiment. A notch filter, a band-stop filter with a narrow stopband, was used to remove the 60 Hz power line noise. To minimize unrelated artifacts throughout EEG recording, each subject was instructed to close his/her eyes and to limit body movement. The EEG signals were amplified using a Polymate AP1532^3^ amplifier and visualized on an APMonitor^4^ prior to filtering with a 0.5–60 Hz bandpass filter. We employed the EEGLAB [15] toolbox to remove major artifacts caused by unintentional body movement and then used the independent component analysis (ICA) functionality of the toolbox to remove eye-movement artifacts.

### DEAP dataset

The DEAP dataset contains EEG and peripheral physiological signals recorded from 32 subjects as they watched 40 selected 1 min excerpts of music videos [10]. In the data collection process, 40 videos were presented in 40 trials, with each trial comprising 2 s of progress display, 5 s of baseline recording, and 1 min of music video watching followed by self-emotion annotation. To self-assess emotional level, each subject rated arousal, valence, dominance, and like/dislike of each music video excerpt on a continuous scale ranging from 1 (low) to 9 (high), and rated familiarity to the music on a discrete scale ranging from 1 (“never heard it before the experiment”) to 5 (“knew the song very well”). EEG signals acquired via 32 electrodes were downsampled to 128 Hz and eye-movement artifacts detected via electrooculography (EOG) were removed. A bandpass filter was applied to extract signals in a frequency range of 4–45 Hz.

## Investigation of EEG correlates of familiarity

One proposal of this study was to investigate EEG correlates underlying feelings of familiarity and unfamiliarity to musical stimuli. As it remains unclear whether music familiarity has any detectable association with EEG signals, we performed two different types of analysis on both our dataset and the DEAP dataset. The first method involved trying to find a familiarity clue in each electrode used in the EEG, while the second one involved examining the links between each of the electrodes.

### Data acquisition

To maximize differences in familiarity and minimize any label ambiguities resulting from the subjective familiarity scores, only the data from the listening session with the most (i.e., familiarity level 6) and least (i.e., familiarity level 1) familiar samples in our dataset were used to perform the analysis. Consequently, we ignored data from subjects 8 and 13, as there was no indication as to which sample had the highest familiarity in their reports. Additionally, we disregarded data from subjects 1 and 3 owing to their reported drowsiness during EEG recording. As subject 12 misunderstood the instruction for familiarity judging, this subject’s data were also discarded.

In the DEAP dataset, familiarity ratings were missing for three subjects, namely subjects 2, 15, and 23. As familiarity was not the main focus in the DEAP experiment and the music videos were selected by the experimenters, the number of music videos with a given level of familiarity differed by subject. In particular, the incidence of reported low familiarity was higher than that of high familiarity. To better balance low and high familiarity sessions, we defined scores 1–2 as low familiarity and 3–5 as high familiarity. However, as imbalance still remained in the data procured from some of the subjects, we also disregarded data from subjects whose high/low familiarity report ratios were less than 0.30. As a result, the data from subjects 4, 5, 25, and 27 were discarded.

### Single-electrode-level power spectral density analysis

For the investigation of the EEG correlates of music familiarity, the power spectral density (PSD) approach, which is based on the fast Fourier transform (FFT), was adopted to obtain the characteristics of brain signals in the frequency domain. In our dataset, the averaged PSDs over the delta (1–4 Hz), theta (4–8 Hz), alpha (8–13 Hz), beta (13–30 Hz), and gamma (30–40 Hz) frequency bands were extracted from all-12-electrode signals using the MATLAB Signal Processing Toolbox^5^. In order to obtain a higher amount of data for analysis, we applied a non-overlapping sliding window segmentation technique in which the window size was defined as 1000 samples, which was equivalent to a 4 s window length (this length corresponds to previous emotion classification work, as will be described in the following section).

Similarly, we decomposed the brain signals in the DEAP dataset into four distinct frequency bands using the PSD approach and extracted the theta (4–8 Hz), alpha (8–13 Hz), beta (13–30 Hz), and gamma (30–40 Hz) bandwaves. It should be noted that, as the preprocessed EEG signals of the DEAP dataset had already been filtered between 4 and 45 Hz, we could not extract the PSD in the delta band. The non-overlapping sliding window technique was also applied, with the window size defined as 512 samples, equivalent to a 4 s window length. However, we found that the PSDs of the signals extracted from some electrodes were oddly high in some subjects; therefore, we regarded any PSD above 100 $\mu V^2/Hz$ as *bad-channel PSD*, as the corresponding signals might have been contaminated by unrelated noises. As a result, more than 25 % of the signals obtained from each of the four subjects, namely subjects 9, 11, 22, and 24 were found to be *bad-channel PSD*; we ignored all data from these subjects and performed analysis using only the data from the other 21 subjects.

#### Statistical analysis

To determine how the PSDs of various bands were affected by music familiarity (high and low) and subject individuality, two-way analysis of variance (ANOVA) with replication was performed. For each frequency band and electrode, we collected multiple PSDs from all subjects and divided them into two groups: low and high familiarity. Replication, i.e., multiple observations, involved obtaining multiple PSDs from each subject. As diversity in song selection and familiarity labeling of each subject produced differences in the number of acquired PSDs, it was necessary to unify the number of replications across subjects. Hence, we defined the number of replications as the minimum size of the available dataset across subjects and familiarity levels, and we aggregated data from each subject by randomly selecting available data up to the replication number. Two-way ANOVA was then performed using MATLAB Statistics and Machine Learning Toolbox^6^ to test the hypotheses that the main effects of familiarity and subjectivity were significant. Post-hoc comparisons were performed using the Tukey test. In testing the DEAP dataset, if a particular subject’s electrode produced *bad-channel PSD* in any frequency band, all PSD data obtained from the electrode were removed before performing ANOVA.

#### Results

**Fig. 1:**
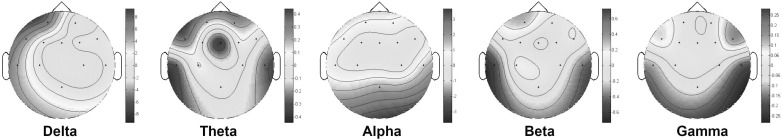
A topological plot of the variation of average PSD values across subjects produced by songs with high and low music familiarity (familiarity power–unfamiliarity power) from our dataset; *positive areas* represent regions in which high familiarity produces higher power than low familiarity, while *negative areas* depict where unfamiliarity produces higher power

**Fig. 2:**
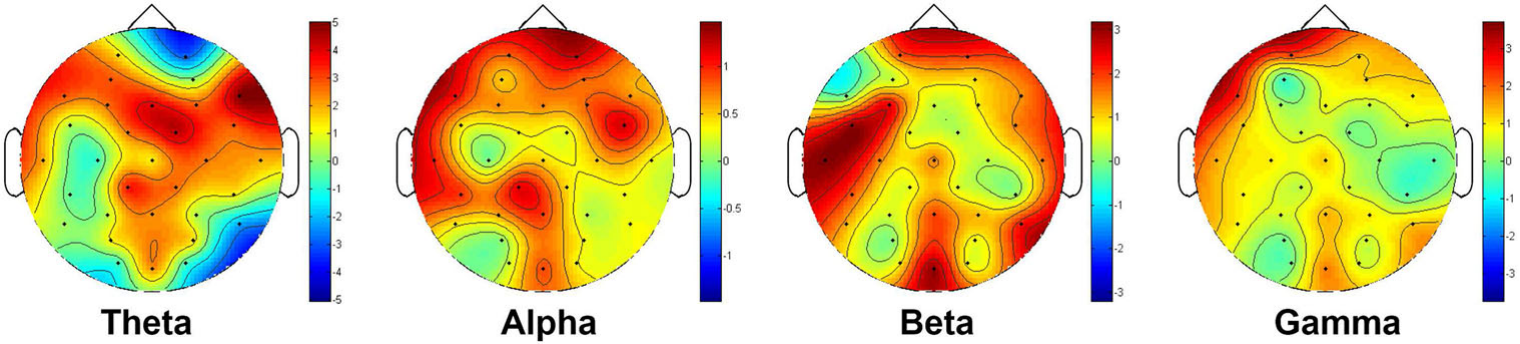
A topological plot of the variation of average PSD value across subjects exposed to music videos with high and low music familiarity (familiarity power–unfamiliarity power) from the DEAP dataset; *positive areas* represent regions in which high familiarity produces higher power than low familiarity, while *negative areas* depict where unfamiliarity produces higher power

We performed ANOVA on our dataset to explore whether there was any significant PSD difference (*p* < 0.05) owing to familiarity. The results showed the main effect of inter-subject variability on variations in PSD values. However, we still found that the familiarity had a statistically significant effect on PSD value, particularly in the frequency bands obtained by some of the electrodes, as shown in Table 1. To investigate further, we calculated the average of the power spectra across subjects under high and low music familiarity and topologically plotted the variation in averages (familiarity–unfamiliarity) on a scalp map, as shown in Fig. 1. On this map, positive areas represent locations where familiar songs evoked higher averaged power spectra across subjects than did unfamiliar songs. Similarly, we performed ANOVA at the significance level *p* < 0.0001 on the DEAP dataset. Again, we found significant variation in PSD values owing to familiarity, as shown in Table 2. The variation in the averaged PSD (familiarity–unfamiliarity) calculated from the DEAP dataset is illustrated in Fig. 2. In the DEAP dataset, the PSD variation owing to familiarity was prominent in the higher frequency bands.

**Table 1:** Significance values *p* from our dataset of the differences between familiar and unfamiliar songs across subjects under single-electrode PSD analysis; emboldened characters emphasize that PSDs taken while listening to music with high familiarity are higher than those taken while listening to music with low familiarity

Band	**Electrodes**	Significant values *p* ($p<$ 0.05)
$\delta$	Fz	0.0005
	**F7**	0.0357
	**T3**	0.0377
$\theta$	Fz	0.0002
$\alpha$	**Fp1**	0.0153
	**Fp2**	0.0260
	**Pz**	0.0292
	**T4**	0.0007
$\beta$	Fz	0.0047
	T3	0.0315
	T4	0.0005
$\gamma$	C4	0.0105
	Pz	0.0003
	**F8**	0.0019
	T4	0.0006

**Table 2:** Significance values *p* from the DEAP dataset of the differences between familiar and unfamiliar music videos across subjects under single-electrode analysis; emboldened characters emphasize that the PSD resulting from watching music videos with high familiarity is higher than that resulting from watching music videos with low familiarity

Band	**Electrodes**	Significant values * p* (*p < * 0.0001)
$\theta$	**CP1**	4.98 × 10^−5^
	**Fz**	1.75 × 10^−5^
	**F8**	5.44 × 10^−6^
	**FC2**	8.66 × 10^−5^
$\alpha$	**F7**	7.95 × 10^−5^
	**CP1**	1.55 × 10^−8^
	**Oz**	6.75 × 10^−5^
	**Fp2**	9.48 × 10^−5^
	**FC6**	4.08 × 10^−7^
$\beta$	**Fp1**	9.62 × 10^−5^
	**FC5**	1.46 × 10^−6^
	**FC1**	6.85 × 10^−8^
	**C3**	5.43 × 10^−6^
	**T7**	8.19 × 10^−8^
	**CP5**	4.23 × 10^−6^
	**CP1**	5.21 × 10^−6^
	**P3**	1.32 × 10^−5^
	**P7**	4.64 × 10^−7^
	**Oz**	4.36 × 10^−10^
	**Pz**	2.78 × 10^−7^
	**AF4**	2.02 × 10^−7^
	**Cz**	3.89 × 10^−9^
	**P4**	7.70 × 10^−6^
	**P8**	1.08 × 10^−5^
	**PO4**	5.39 × 10^−8^
	**O2**	3.37 × 10^−6^
$\gamma$	**Fp1**	1.03 × 10^−6^
	**T7**	1.91 × 10^−5^
	**P3**	2.29 × 10^−6^
	**P7**	4.08 × 10^−9^
	**Oz**	1.81 × 10^−8^
	**AF4**	5.16 × 10^−15^
	**Cz**	9.16 × 10^−9^
	**P8**	4.81 × 10^−5^
	**PO4**	8.50 × 10^−5^
	**O2**	1.63 × 10^−7^

It was previously discovered that listening to unfamiliar songs relates to recollection, the cognitive ability to recall a former context associated with a musical excerpt by utilizing *episodic memory* [16]. We hypothesized that subjects in our experiment might recollect past experience from episodic memory to identify a novel song. Previous research [17] that showed relatively higher gamma power over the parietal scalp during the act of recollection (as opposed to the act of experiencing familiarity) is consistent with our results that showed a marginally higher gamma-PSD obtained from the Pz electrode while listening to an unfamiliar song. In addition, Hsieh and Ranganath [18] also reported on the implication of the frontal midline $\theta$ in working and episodic memory in which the associated memories could possibly be relevant to unfamiliar song listening. However, subjects in the DEAP experiments produced higher gamma and frontal midline theta power while watching familiar music videos; we suspect that the underlying reason for this is that the subjects used memory to a greater extent to anticipate the next scene of a music video because they might have occasionally watched the music video versions of regularly listened to songs. Unlike our dataset, subjects in DEAP dataset experiment who watched a particular music video for the first time or who had minimal experience with the video would engage so intensely enough in watching the video that they avoided using any recollection memory to associate the music with previous experiences. This evidence indicated that familiarity to video scenes had a higher influence on brain activities than familiarity with the music used as background sounds in the music video.

Moreover, the increase in Fz theta power in our results corresponds with the previous reports of enhancement of frontal midline theta rhythm (Fm$\theta$) during focused attention [19]. A likely underlying reason for this is that song unfamiliarity induced our subjects to listen more attentively in order to successfully annotate emotions subsequently in the following phase.

### Functional connectivity analysis

As most brain functions have been shown to involve multiple brain sites rather than a single specific site, EEG-based analysis of brain activity at the level of interrelation between electrode pairs can offer deeper insights into the association between brain activity and music familiarity. In addition to the above-described analysis at the single-electrode level, we performed an investigation of brain functional connectivity in association with music familiarity. To perform analysis in specific EEG frequency bands, we applied a fifth order bandpass Butterworth filter to extract EEG signals in the delta, theta, alpha, beta, and gamma frequency bands from our dataset and to extract EEG signals in theta, alpha, beta, and gamma frequency bands from the DEAP dataset. As in the single-electrode-level analysis, we analyzed only valid data from the 10 subjects in our dataset and from the 21 subjects in the DEAP dataset. We then calculated connectivity indices from all pairs of electrodes independently in each frequency band using the three following approaches, which have been commonly employed in many studies of EEG correlates, including studies of the neural correlates of emotion [20]. These three connectivity indices have been demonstrated to be sensitive to different characteristics of EEG signals.

*Correlation* corresponds to the relationship between two signals from different brain sites. Given signals *x* and *y*, the correlation at each frequency (*f*) is a function of cross-covariance $C^f_{xy}$ and auto-covariances, $C^f_{xx}$ and $C^f_{yy}$, of *x* and *y*:

1 $$R_{xy}(f) = \frac{C^f_{xy}}{\sqrt{C^f_{xx}C^f_{yy}}}.$$

*Coherence* is similar to correlation that also includes the covariation between two signals as a function of frequency. This index indicates how much two brain sites are working closely together at a specific frequency band. Given signals *x* and *y*, coherence is a function of the respective power spectral densities, $P_{xx}(f)$ and $P_{yy}(f)$, of *x* and *y*, and of the cross-PSD, $P_{xy}(f)$, of *x* and *y*:

2 $$Coh_{xy}(f) = \frac{\bigm |P_{xy}(f)\bigm |^2}{P_{xx}(f)P_{yy}(f)}.$$

*Phase synchronization index (PSI)* is a non-linear measure of connectivity. The PSI among brain regions indicates connectivity in terms of the phase difference between two signals. PSI can be restricted to certain frequency bands reflecting specific brain rhythms. For two signals *x* and *y* with data length *L*, the PSI is defined as

3 $$PSI_{xy}=\left |\frac{1}{L}\sum _{t=0}^{L}e^{i[\phi _x(t)-\phi _y(t)]}\right |,$$

where $\phi _x(t)$ = $\arctan \tilde{x}(t)\mathbin {/}x(t)$ is the Hilbert phase of signal *x* and $\phi _y(t)$ is the phase of signal *y*, while $\tilde{x}(t)$ is the Hilbert transform of *x*(*t*).

#### Statistical analysis

The results of the single-electrode-level analysis showed that inter-subject variability affected brainwave disparity to a much greater degree than music familiarity. Unlike this analysis at the single-electrode level, in which we retrieved multiple data for statistical analysis from one subject, in the multiple electrode analysis, we calculated a single functional connectivity index for each subject to represent overall brain connectivity in each electrode pair in each frequency band. In other words, a single connectivity index was derived from EEG signals produced for each subject-song pair. Then, the connectivity indices were separated into two groups in accordance with music familiarity (low and high), and a unified index was calculated to represent the overall index for all subject-song pairs in each familiarity group. Because coherence and PSI range from 0 to 1 and correlation ranges from –1 to 1, we calculated the arithmetic mean to derive the overall coherence and PSI, and the quadratic mean to derive the overall correlation across songs. We then performed paired *t-test* using the MATLAB Statistics and Machine Learning Toolbox to discover any statistically significant difference in EEG functional connectivity associated with music familiarity across subjects.

#### Results

**Fig. 3:**
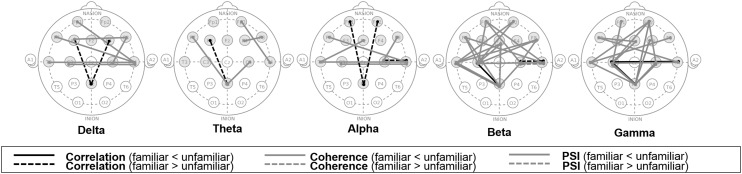
Functional connectivity with significant difference values (*p* < 0.05) owing to music familiarity from our dataset; *lines* indicate significantly higher (*solid*) and lower (*dash*) connectivity indices resulting from listening to unfamiliar songs as compared to listening to familiar songs

**Fig. 4:**
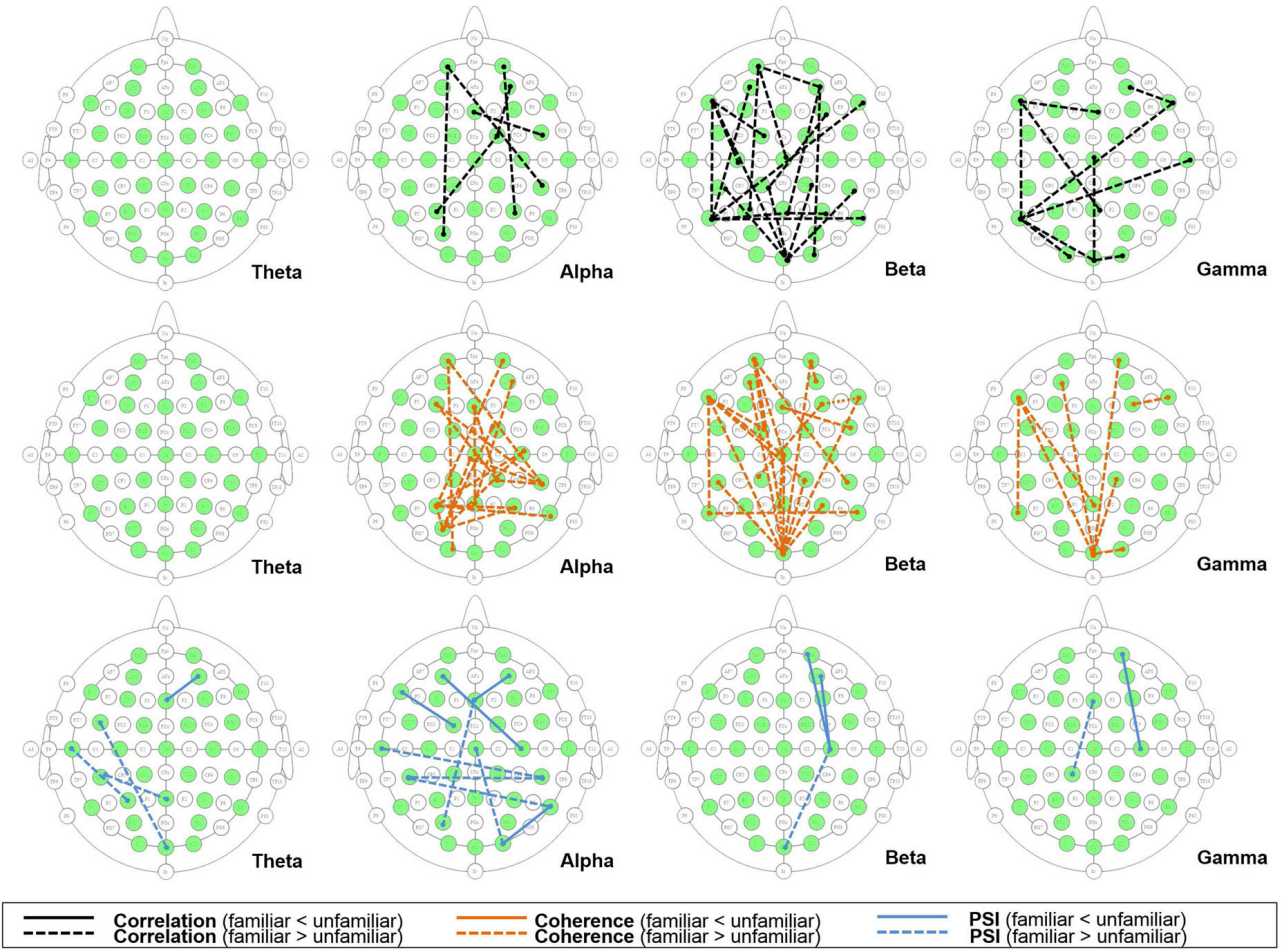
Functional connectivity with significant difference values (*p* < 0.05) owing to music familiarity from the DEAP dataset; *lines* indicate significantly higher (*solid*) and lower (*dash*) connectivity indices when listening to unfamiliar songs as compared to listening to familiar songs

The significant variations in functional connectivity were mapped to a scalp map, as illustrated in Figs. 3 and 4. From our dataset, we discovered an increase in connectivity, especially in the higher frequency bands, when subjects listened to unfamiliar songs. Burgess and Ali [17] reported greater functional connectivity in the gamma band during an experience of recollection compared to that during an experience of familiarity. Our results agree with this study, as we found higher connectivity resulting from unfamiliar songs, especially in the gamma frequency range. Imperatori et al.  [21] found higher delta and gamma band connectivity during the performance of autobiographical memory tasks. In light of our hypothesis regarding episodic memory use during unfamiliar song listening, our results were consistent with their findings. Additionally, we found an increase of connectivity in the DEAP dataset, especially in higher frequency bands, when the subjects watched familiar music video excerpts. This phenomenon is probably related to cognitive recollection, and hypothesized use of episodic memory to anticipate the next video scene might be the underlying cause.

Interestingly, the correspondence between single-electrode-level analysis and functional connectivity analysis might confirm that music familiarity elicits detectable changes in brain activities that probably relate to memory recollection.

## Familiarity effects in emotion recognition systems

In the previous section, we demonstrated that music familiarity affects EEG signals using both analysis at the single-electrode level and the functional connectivity level. In this section, we present the results of EEG-based emotion recognition assessment that takes music familiarity into account. To measure this, we separated EEG signals into two groups in accordance with familiarity level (low and high). In our dataset, we separated the data from songs into a high familiarity data group (4–6 familiarity scores) and a low familiarity data group (1–3 familiarity scores). For the DEAP dataset, we used the same separation approach as in the previous section. Features were then separately extracted from the EEG signals of each data group and used to train emotion recognition models. As a comparison with the traditional approach that overlooks the familiarity effect, we also trained a model to use features extracted from all data groups (i.e., the original data before separation).

### Feature extraction

The fractal dimension (FD) value reveals the complexity of a time-varying EEG signal and has been recently used in affective computing research, including studies of EEG-based emotional state estimation [22]. A higher FD value for an EEG signal reflects higher activity in the brain [23]. The FD approach is appealing because of its simplicity and ability to informatively reveal characteristics that can properly indicate a variety of brain states. In this study, we derived the FD value by using the Higuchi algorithm [24].

We also extracted PSD data to characterize EEG signals in the frequency domain, which has become a common practice in the estimation of emotional states [3]. We used the same PSD ranges as those used in the previous section as features for emotion classification model training.

A review of literature on the subject of using DEAP datasets reported that the best emotion classification results could be obtained by using a sliding window size of 3 s for arousal classification and 6 s window size for valence classification in the feature extraction process [25]. For the sake of simplicity, in this work, we applied a 4 s sliding window without overlapping between consecutive windows for both arousal and valence classification in order to retrieve a higher amount of data points from each song/video. Using timestamps, we labeled each instance with an associated ground-truth emotion. In our dataset, we used a *majority* approach to determine the associated emotional label for each particular window containing variation in emotion annotation. In the DEAP dataset, multiple extracted features from each video were labeled using the single emotion reported by each subject.

The asymmetries of features in spatially symmetric electrode pairs were taken into account in this study, as such hemispheric asymmetries have been shown to be informative in classifying emotions in previous research [10, 22, 26]. An additional differential asymmetry feature was calculated by subtracting a feature in the right-hemisphere electrode’s signal from the same feature extracted from the signal produced by the symmetric electrode in the left hemisphere. We obtained additional features from our dataset from five symmetric electrode pairs throughout the brain and from 14 symmetric electrode pairs in the DEAP dataset. In total, 17 FD and 85 PSD features were extracted from our dataset, while 46 FD and 184 PSD features were extracted from the DEAP dataset.

### Emotion classification

Emotion recognition was converted into a binary classification by separating arousal into high and low classes and valence into positive and negative classes. Each class in our dataset was determined by the positivity of arousal and valence ratings. In the DEAP dataset, the instances were classified into the high arousal class when arousal rating was higher than 4.5; otherwise, they were placed in the low arousal class. Similarly, the data with a valence rating of above 4.5 were placed in the positive valence class, and the other data points were placed in the negative valence class.

We used the WEKA [27] library to apply three commonly used algorithms to classify emotional classes: a support vector machine (SVM) based on the Pearson VII kernel function (PUK) kernel, a multilayer perceptron (MLP) with one hidden layer, and C4.5. The overall performance of emotion recognition within each subject was evaluated using the 10-fold cross-validation method. As we relied on self-annotation from subjects, the imbalance of datasets has misled us in the interpretation of results; correspondingly, we defined a new baseline—the *chance level* or percentage of data points in the majority class. For instance, a dataset from a subject comprising of 60 % positive and 40 % negative arousal samples would have a chance level of 60 %. In each subject’s data group, the results of classification were compared to the chance levels in order to evaluate the performance of emotion recognition relative to that of the majority-voting classification.

### Results of emotion classification

As described in the previous section, data from three subjects were removed from our dataset owing to reports of drowsiness and instruction misunderstanding. We then classified data from these remaining 12 subjects. The averaged confidence level of correspondence in annotation across these remaining subjects was 2.4063 ($SD = 0.6565$), which indicated that the annotated data in our dataset were applicable. We also classified the data produced by the remaining 21 subjects in the DEAP dataset.

**Fig. 5:**
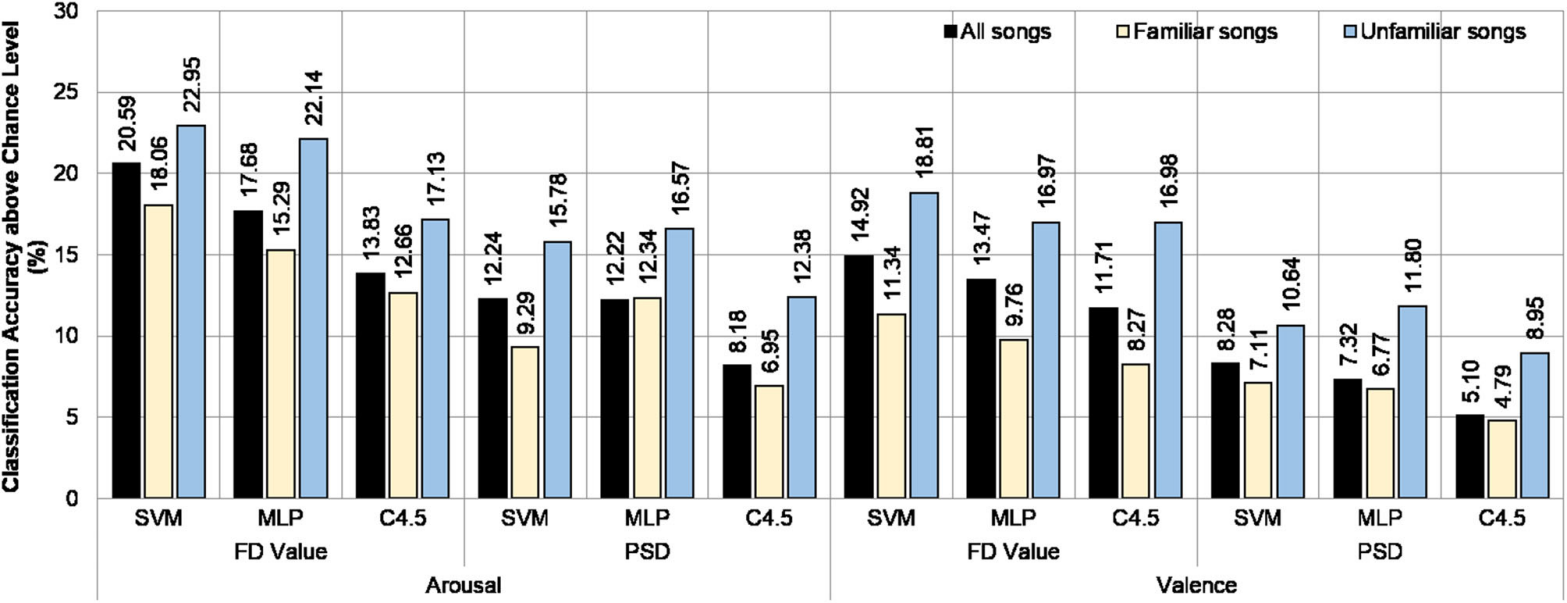
Arousal and valence classification accuracies above the chance levels for high familiarity (*familiar songs*), low familiarity (*unfamiliar songs*), and combined (*all songs*) data groups from our dataset

The classification accuracies above the chance levels averaged over the subjects from our dataset are shown in Fig. 5. In arousal recognition, the degree of classification above the chance level using only data from unfamiliar song sessions was superior to that using the overall dataset, and the data from familiar song sessions achieved the lowest performance. The best results were obtained by classifying FD features with SVM using unfamiliar song data, which achieved 87.80 % ($SD = 7.73\, \%$) averaged accuracy against a chance level of 64.86 % ($SD = 7.04\, \%$). Similarly, valence recognition using unfamiliar song data provided better results than using familiar song data or the total dataset. Again, classifying FD features using SVM produced the highest relative accuracy: 86.91 % ($SD = 8.13\, \%$) averaged absolute accuracy against a chance level of 68.10 % ($SD = 11.79\, \%$). However, the results of a statistical *t-test* indicated that the superiority of using unfamiliar data over other types of data in emotion classification was not statistically significant.

**Fig. 6:**
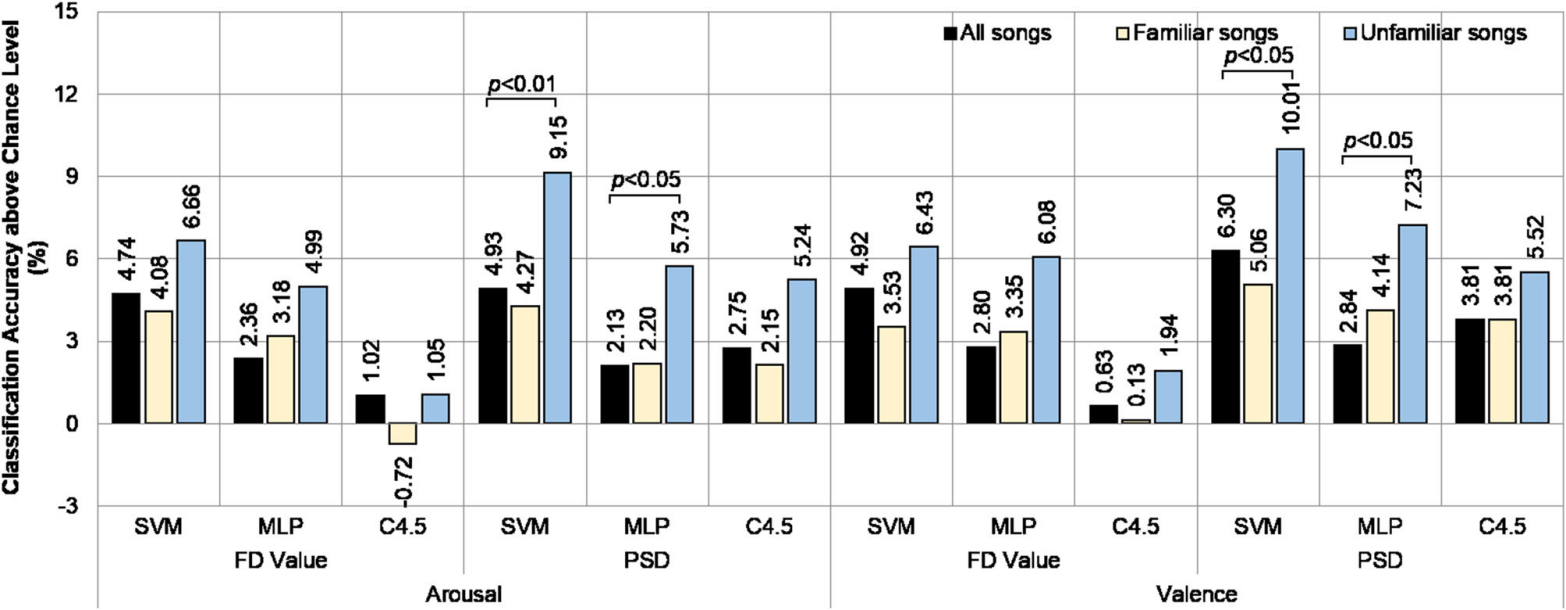
Arousal and valence classification accuracies above the chance levels for high familiarity (*familiar songs*), low familiarity (*unfamiliar songs*), and combined (*all songs*) data groups from the DEAP dataset

Figure 6 shows the averaged classification accuracies over the chance levels across subjects using the DEAP dataset. Similar to the results obtained using our dataset, classifying arousal and valence by using data from unfamiliar music video sessions achieved higher performance than by using either high familiarity sessions or the overall dataset. In arousal recognition, the best result over the chance level was obtained by classifying PSD features with SVM using data from low familiarity sessions; this methodology achieved 73.30 % ($SD = 7.71\, \%$) averaged accuracy across subjects against a chance level of 64.15 % ($SD = 10.70\, \%$). In valence recognition, using PSD features extracted from EEG signals in low familiarity sessions to classify using SVM achieved the highest relative performance, with an absolute performance of 72.50 % ($SD = 6.91\, \%$) against a chance level of 62.49 % ($SD = 8.02\, \%$). Furthermore, statistical *t-test* revealed that classifying PSD features with either SVM or MLP using data from low familiarity music video sessions were significantly better than classifying by the same approach using the overall dataset.

The superior performance of SVM relative to other algorithms has also been shown in the previous studies [3]. This superiority can be attributed to SVM’s better capability for analyzing the non-linear behaviors of the brain.

## Discussion

Our EEG-correlate evidence reveals that the effects of familiarity are reflected in brain activities measured through PSD results and brain functional connectivity studies. However, the effectiveness of emotion recognition using EEG might suffer if the subject’s familiarity with the musical stimuli is disregarded. Experiments using both our dataset and the DEAP dataset came to the consistent conclusion that data from sessions using only unfamiliar musical excerpts provide better EEG-based emotion classification than data using familiar musical excerpts or a combination of both data types. In summary, the empirical results of our emotion recognition study suggest that unfamiliar musical stimuli might be the most appropriate material to evoke emotion in the construction of an emotion recognition system. In addition, experiencing unfamiliar musical stimuli would also eliminate the factors of expectation and predictability that have been reported to influence emotional response to music [8].

One of the major differences between our dataset and the DEAP dataset is the approach to annotation. Our EEG experiments allowed subjects to continuously report emotion in arousal-valence space; by contrast, subjects who produced the DEAP dataset could report only one perception for each music video watched. The temporal continuity of emotion reporting in our experiments led to a higher granularity in emotion capturing compared to the DEAP dataset, which could be the underlying reason why the emotion recognition using our dataset had achieved higher performance over the chance level than that using the DEAP dataset.

Another difference between the two datasets was the stimuli used. In our dataset, MIDI files were used and subjects were instructed to close their eyes while listening to the music. By contrast, the experiments producing the DEAP dataset used music videos and the subjects kept their eyes open to watch these. According to our results, the FD approach could achieve better performance in terms of emotion classification than PSD, whereas the PSD performed better for the DEAP dataset. The superiority of the PSD to the FD approach in EEG-based emotion recognition was also seen in previous work using music videos [28] and movie clips [29] as stimuli. To the best of our knowledge, although FD features have been found to be successful in emotion recognition when using music as stimuli [22], none of the previous works directly compared performance in terms of music-emotion recognition between FD and PSD features. This study, therefore, provides an initial of music-emotion classification comparison between the use of FD and PSD features. The actual association between stimuli difference and classification results is a subject worthy of systematic investigation in a dedicated study, which we propose to conduct in future work. In addition, as the DEAP dataset produced variations in PSD that most prominently appeared in higher frequency bands, which are related to high cognitive functions, we are encouraged to further study whether the cognitive level has any influence on familiarity and its related processes.

Despite the novel results of the study discussed in this paper, the mechanisms underlying the effects of music familiarity on brainwaves remain unclear and are worthy of further investigation. Extending the present study by including more subjects or using another sophisticated analysis tool such as event-related potential to validate the current findings is another prospective area for our future work. In addition, incorporating familiarity information into the process of building an emotion classifier can possibly improve the performance of emotion estimation, which represents yet another avenue for future work.

## Conclusions

This study presented evidence for the association between EEG signals and music familiarity based on the analysis of single-electrode-level PSD and brain functional connectivity. We demonstrated that classifying emotion using typical algorithms can benefit from controlling the familiarity level of the subject to musical stimuli. In particular, using data collected solely from unfamiliar stimuli perception can help achieve more accurate emotion classification results, which suggests that unfamiliar musical stimuli are more appropriate for use in the construction of emotion recognition systems.

## Footnotes

1
http://docs.oracle.com/javase/7/docs/technotes/guides/sound/.

2
http://www.ant-neuro.com/products/waveguard.

3
http://www.teac.co.jp/industry/me/ap1132/.

4 Software developed for Polymate AP1532 by TEAC Corporation.

5
http://www.mathworks.com/products/signal/.

6
http://www.mathworks.com/products/statistics/.
